# An Unusual Suspect Causing Hypoxemic Respiratory Failure

**DOI:** 10.1177/2324709616687587

**Published:** 2017-01-01

**Authors:** Masooma Aqeel, Bjorn Batdorf, Horatiu Olteanu, Jayshil J. Patel

**Affiliations:** 1 Medical College of Wisconsin, Milwaukee, WI, USA

**Keywords:** ARDS, respiratory failure, hypoxemia, lymphoma, antisynthetase syndrome

## Abstract

**Introduction:** Antisynthetase syndrome (ASS) is characterized by the presence of anti-Jo-1 antibodies in conjunction with clinical findings of fever, polymyositis-dermatomyositis, and interstitial lung disease (ILD). Inflammatory myopathies carry a high risk of malignancy, but this association is less well outlined in ASS. We present the case of a patient with ASS who developed non-Hodgkin’s lymphoma with acute hypoxemic respiratory failure. **Case Presentation:** A 44-year-old female with ASS presented with acute hypoxemic respiratory failure. She was empirically treated with broad-spectrum antibiotics for a health care–associated pneumonia; however, she failed to improve. Chest computed tomography revealed extensive bilateral ground glass opacities as well as extensive mediastinal and axillary lymphadenopathy. Infectious workup was negative. A surgical lung biopsy revealed peripheral T-cell lymphoma (PTCL). The patient was started on chemotherapy with complete resolution of hypoxemic respiratory failure. **Conclusions:** Malignancy is very rare in the setting of ASS; and our case illustrates the unique presentation of PTCL in ASS. In addition, lung involvement in PTCL is variable (incidence ranging from 8% to 20%); and in this case, bilateral multifocal consolidation was biopsied and proven to be PTCL involving the lungs. This case highlights the rare noninfectious conditions that can present as acute hypoxemic respiratory failure in the setting of ASS.

## Introduction

Antisynthetase syndrome (ASS) is a systemic autoimmune disorder characterized by the presence of antibodies against amino acyl-transfer RNA synthetases found in conjunction with fever, “mechanic-hands,” polymyositis-dermatomyositis, Raynaud’s phenomenon, arthritis, and/or interstitial lung disease (ILD).^1-3^

These antibodies target and impair the function of cytoplasmic enzymes (aminoacyl-transfer RNA [t RNA] synthetases [ARS]), which are responsible for the formation of amino-acyl-t RNA complexes during the translational phase of protein synthesis. Eight such antibodies have been discovered to date,^2^ and of these, the anti-histidyl-aminoacyl-t RNA synthetase (anti-Jo-1) antibodies are probably the most clinically important ones.^4,5^ Approximately a third of patients with inflammatory myositis are anti-Jo-1 positive, and evidence suggests that anti-Jo-1 antibody levels correlate directly with severity of muscle and lung disease activity in ASS.^6^

The pathogenic mechanisms by which these antibodies act against the protein synthesis machinery and trigger an autoimmune response are not completely understood and are under investigation. One such proposed hypothesis states that fragments of t RNA synthetase may have cytokine-like effects and may trigger release of tumor necrosis factor-α (TNF-α) from macrophages. Another hypothesis suggests that anti-Jo-1 antibodies may directly upregulate major histocompatibility complexes causing muscle and lung inflammation in murine models.^1^

Inflammatory myopathies like polymyositis and dermatomyositis may present alone or as part of larger “antisynthetase syndrome.” An independent association between polymyositis and/or dermatomyositis and malignancy is well established in medical literature.^7-9^ However, despite the association between autoimmune myopathies and a risk for malignancy, there is no known association between ASS and malignancy. We present a rare case of malignancy presenting as acute hypoxemic respiratory failure in a woman with ASS.

## Case Report

A 44-year-old woman with an 18-year history of ASS and rheumatoid arthritis (RA) overlap complicated by 5 years of nonspecific interstitial pneumonitis (NSIP) presented to the emergency department with 2 weeks of progressively worsening dyspnea and cough. The patient was diagnosed with polymyositis via muscle biopsy approximately 20 years prior. At 38 years of age, she developed progressively worsening exertional dyspnea. Anti-Jo-1 antibodies were positive, confirming a diagnosis of ASS. Chest computed tomography (CT) revealed bilateral subpleural ground glass reticulation and fibrosis, consistent with NSIP. A transbronchial lung biopsy also confirmed NSIP. Current immunosuppressive regimen included tacrolimus and hydroxychloroquine for ASS.

**Figure 1.**
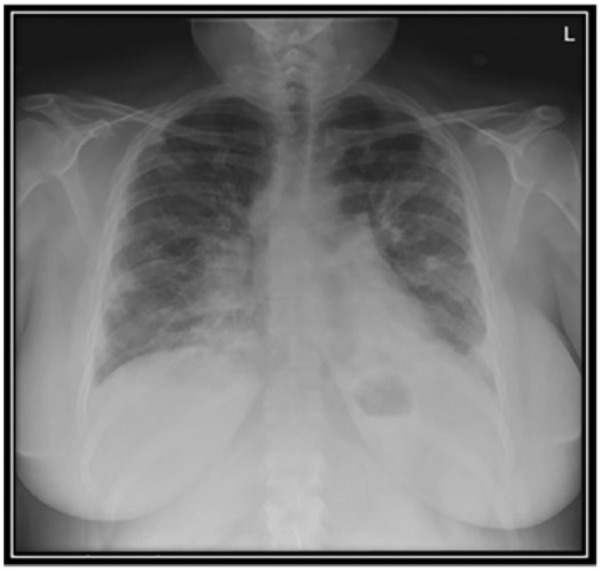
Chest X-ray: acute onset of diffuse bilateral alveolar and interstitial opacities.

Initial blood pressure was 149/59 mm Hg, heart rate of 103/minute, respiratory rate of 26/minute, temperature 38.2°C, and pulse oximetry (SpO_2_) 79% on room air. The patient appeared to be in mild to moderate respiratory distress and had coarse bi-basilar crackles. Admission chest radiography showed new diffuse bilateral patchy alveolar and interstitial opacities (Figure 1). Initial laboratory data showed a white blood cell count of 24 500/mm^3^ (38% neutrophils and 54% lymphocytes), a hemoglobin of 12.2 g/dL, platelet count of 268 000, blood urea nitrogen 26, creatinine 1.01 mg/dL, and potassium 5.0 mEq/L. Initial differential diagnoses included the adult respiratory distress syndrome (ARDS) from infection versus an acute exacerbation of ILD.

She was admitted to the intensive care unit and initially supported with high-flow oxygen via nasal cannula. Empiric antimicrobials targeting health care–associated pathogens and influenza were started for a working diagnosis of infectious pneumonia-induced ARDS in an immunocompromised host.

Urine *Streptococcus*, *Legionella*, *Histoplasma*, and *Blastomycosis* antigens were negative. Sputum gram stain and culture, sputum fungal culture, respiratory influenza nucleic acid amplification test (NAAT), respiratory viral NAAT, serum cytomegalovirus NAAT, *Pneumocystis jiroveci* direct fluorescent antibody, and serum *Aspergillus* galactomannan antigen were all negative.

**Figure 2.**
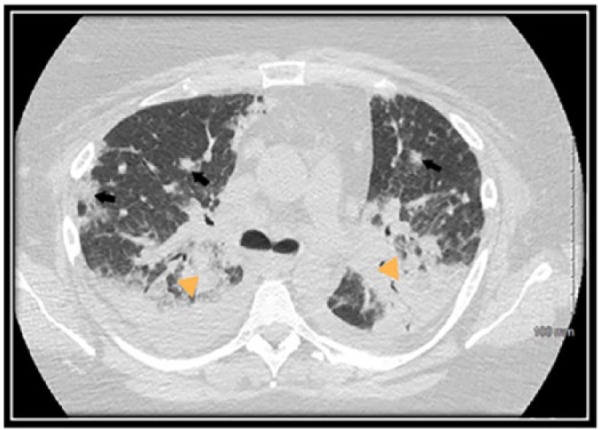
Chest computed tomography scan on admission demonstrating diffuse nodular opacities with spiculated peripheral ground opacities (arrows) and bilateral lower lobe consolidation (arrow heads).

**Figure 3.**
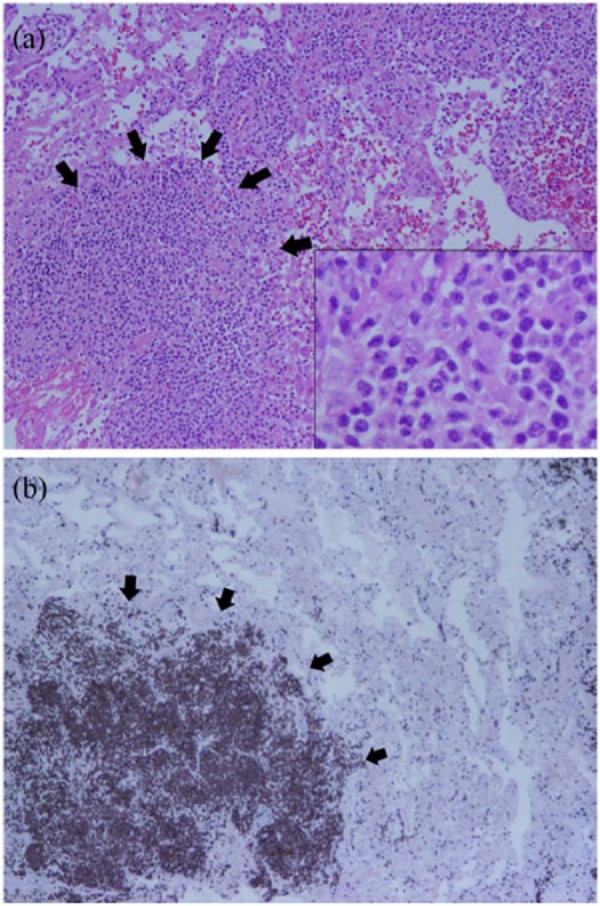
(A) Surgical lung biopsy (hematoxylin and eosin, 40× magnification) demonstrating lung parenchyma with focal dense lymphoid infiltrates (black arrows). Inset (100× magnification) composed of medium-sized cells with irregular nuclear contours, variably condensed chromatin, inconspicuous nucleoli, and moderate amount of cytoplasm. (B) Surgical lung biopsy (CD3 immunohistochemistry, 40× magnification) demonstrating a neoplastic aggregate of CD3 (+) cells (black arrows). The lymphoma cells were also positive for CD4, CD5, and CD7 and negative for CD8, CD30, ALK-1, and EBER.

**Figure 4.**
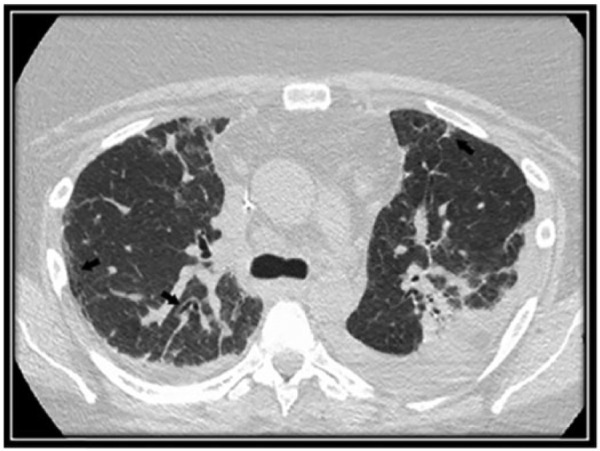
Chest computed tomography scan after therapy. Resolution of nodular infiltrates with residual traction bronchiectasis and fibrosis from known nonspecific interstitial pneumonitis.

On hospital day 2, chest CT revealed bilateral patchy consolidation and multiple enlarged axillary and mediastinal lymph nodes (Figure 2). On hospital day 3, her hypoxemia and clinical condition continued to decline necessitating endotracheal intubation. Bronchoscopy for airway examination was unremarkable. Bronchoalveolar lavage for infectious testing was also negative. On hospital day 5, video-assisted thoracoscopic biopsies were taken from the right middle and lower lobes. Histopathology revealed peripheral T-cell lymphoma, not otherwise specified (PTCL-NOS; Figure 3A and B). Staging workup revealed stage 4 disease. On hospital day 10, a chemotherapeutic regimen of cyclophosphamide, doxorubicin, vincristine, and prednisone was started. Chest CT revealed interval improvement in pulmonary infiltrates and patchy consolidation (Figure 4), and the patient was extubated on hospital day 13 and discharged home on hospital day 28 with 3 liters per minute oxygen.

## Discussion

Our case highlights 2 rare and important findings. First, our case highlights malignancy in the setting of ASS. Second, it highlights the presentation of PTCL-NOS as an acute hypoxemic respiratory failure in a patient with ASS.

As described earlier, inflammatory myopathies (particularly dermatomyositis) carry a high risk for malignancy. In a pooled analysis of 618 patients from Sweden, Denmark, and Finland, Hill et al demonstrated a higher risk for certain cancers (eg, ovarian, lung, colorectal) in patients with polymyositis and/or dermatomyositis.^7^ Large population-based cohort studies have shown that having dermatomyositis, in particular, is associated with a 6- to 7-fold increased risk for malignancy (standardized incidence risk = 7.7) while polymyositis is associated with an approximately 2-fold increased risk for malignancy.^8-10^ The literature speculates that inflammatory myopathy may represent a *paraneoplastic* manifestation of the underlying malignancy.^10^

While independently existing inflammatory myopathies carry a higher risk for malignancy, they do not pose a higher risk for malignancy when part of the ASS (ie, in the presence of anti-Jo-1 antibodies). There is a paucity of case reports of malignancy in ASS (Table 1) and there are no clear associations between ASS and risk for malignancy. On the contrary, reports suggest the presence of anti-Jo-1 antibodies may have a protective effect for malignancy in patients with ASS.^11^ Other factors, such as the presence of ILD in patients with cancer-associated myositis, may also confer a protective role against malignancy.^10,12^

**Table 1.** Published Cases of Concomitant Malignancy Diagnosed After Diagnosis of Antisynthetase Syndrome.

Title	Authors	Year Published	Case Description
A case of interstitial pneumonitis associated with polymyositis complicated by renal cell carcinoma	Iwasaki et al^13^	1992	A 55-year-old woman found to have an interstitial pneumonitis concomitantly with a renal mass (biopsy showed renal cell carcinoma). Six months after a diagnosis of cancer, she also tested positive for anti-Jo-1 antibodies and diagnosed with antisynthetase syndrome.
Jo-1 syndrome with associated poorly differentiated adenocarcinoma	Watkins et al^14^	2004	A 58-year-old male smoker presented with a dry cough, dyspnea, and clubbing; and was diagnosed with antisynthetase syndrome. Subsequently the patient acutely declined and imaging revealed extensive lymphadenopathy and recurrent thromboses. Lymph node biopsy revealed poorly differentiated adenocarcinoma. The patient had an excellent response to broad antitumor range cytotoxic therapy against colon, gastric and lung cancer.
Hodgkin’s lymphoma in a patient with Jo-1 syndrome	Adam et al^15^	2007	A 47-year-old male with myalgias, exertional dyspnea and fatigue, bilateral ground-glass opacities on imaging. He was diagnosed with antisynthetase syndrome and improved on immunosuppressive therapy. Three years later he presented with extensive lymphadenopathy that was biopsied to show classical Hodgkin’s lymphoma.

Our patient had a positive RA factor and was treated with TNF inhibitors—both of which have been suggested to increase risk of lymphoma.^16,17^ Proposed mechanisms for this association includes dysregulated immunity and chronic immunosuppression in RA, which disables the immune system from surveying and suppressing neoplastic lymphoid cell line and virus growth.^18^ Similarly, TNF-α produces a variety of cellular responses that could be both pro- and anti-tumorigenesis.^19^ Recent data suggest that the *severity* of disease activity in RA, as opposed to TNF-inhibition per se, leads to an increased risk for lymphomas.^17,20^ Reports on these risks are still conflicting, and to date, there is no established cause-effect relationship. Anti-Jo-1 antibodies may play a role in modulating host immunity to affect tumorigenesis. Until these mechanisms are clearly delineated and better understood, we speculate development of lymphoma in our patient was likely a combination of complex pathogenic mechanisms.

Second, our case describes a rare presentation of a rare disease. Our patient presented with hypoxemic respiratory failure with bilateral patchy opacities and was diagnosed with PTCL. PTCL-NOS remains a rare disease and carries a poor overall prognosis with a 5-year survival of only 32%.^21^ In 1992, incidence was 0.1 per 100 000 individuals, and in 2006, it increased to 4 cases per 100 000 individuals.^22^ In developed countries, PTCL-NOS is the most common subtype of PTCL, accounting for 5% to 20% of all non-Hodgkin’s lymphomas. Overall, lung involvement in PTCL is variable, with incidence ranging between 8% and 20%.^23,24^ Lung disease in PTCL-NOS has been radiographically described as diffuse ground-glass opacities,^24^ mass-like consolidation,^25^ pulmonary nodules,^26^ and/or an indolent progression of subacute to chronic ILD.^24,27-30^ Our patient also had mediastinal lymph node enlargement, which is more commonly described for Hodgkin’s lymphoma rather than non-Hodgkin’s lymphomas.^28^ Despite these varied presentations, an acute and fulminant respiratory failure (ARDS) from PTCL is an exceedingly rare occurrence. Certain patterns of pulmonary parenchymal involvement with lymphoma may be associated with a particularly poor prognosis (ie, an alveolar pattern)^28^ but in general it is not known whether pulmonary involvement in PTCL itself signifies a poor prognosis. To our knowledge, there are only 3 case reports of rapidly progressive or relapsing lymphoma presenting as hypoxemic respiratory failure with bilateral infiltrates.^31-33^ Our patient presented with acute hypoxemic respiratory failure and was found to have diffuse bilateral infiltrates. Initial and repeat infectious workups were negative, and the patient had persistent bilateral infiltrates despite empiric antibacterial therapy. On PTCL diagnosis, our patient received chemotherapy with subsequent resolution of hypoxemic and bilateral infiltrates (Figure 3).

In conclusion, our case highlights several important clinical points. First, the identification of a hematological malignancy in ASS is rare and there are conflicting reports over the role anti-Jo-1 antibodies in the evolution of a malignant process. Second, clinicians should be aware of the ever-increasing armamentarium of therapies (such as TNF inhibitors) in chronic inflammatory disease states (such as RA or inflammatory myopathies), and that these together may pose a cumulative risk for malignancy. Third, although rare, PTCL may present as diffuse bilateral patchy opacities. To our knowledge, ours is the first case of PTCL-NOS presenting with acute hypoxemic respiratory failure with diffuse bilateral opacities in a patient with ASS.
